# A Case of Accidental Swallowing of a Cent of the Old Coinage, &c.

**Published:** 1858-06

**Authors:** O. C. Gibbs

**Affiliations:** Frewsburg, Chaut. Co., N. Y.


ART. III.—*A Case of Accidental Swallowing of a Cent of the Old Coinage, &c.* By O. C. Gibbs, M. D., Frewsburg, Chaut. Co., N. Y.

The following cases of accidental swallowing of foreign substances, may not be altogether uninteresting as additions to those presented in the October number of your Journal for 1857. As the cases, with one exception, did not come under my observation, but were verbally reported to the Medical Society of South Western New York, at its last session, Feb. 3d, 1858, this paper is written from memory.

*Dr. Andrews* reported as follows:

About a year ago, I was called to see a lad about five years of age who, it was said, had swallowed a cent of the old coinage. The patient did not seem to be suffering much, but on trial it was found that he could not swallow solid food. On examination, the cent was found lodged low down in the œsophagus, and was carried to the stomach by the aid of a probang. A

laxative dose of castor oil was given. The friends were requested to watch the alvine evacuations, and inform me if at any time unpleasant symptoms should arise. Two weeks after this I was called to see the patient laboring under dysenteric symptoms. These symptoms yielded to opiates and astringents. Having learned that the friends of the patient had been injudiciously using cathartics, with the view of facilitating the escapement of the coin, as well as with the view of carrying off the poisonous products of chemical decomposition, I advised that no medicine should be given except at my direction and to meet present symptoms.

About *six months* from the time the coin was swallowed, it was passed per anum. The boy's health was rather delicate during the period of the retention of the coin, but not sufficiently so to call for any decided treatment. The health is now perfectly restored.

I look upon this case as remarkable in the limited amount of disturbance which the coin occasioned, and in the length of time which it was retained. I have no remembrance of having read of a case where a copper coin was so long retained.

*Dr. O. C. Gibbs* remarked, that in the Buffalo Medical Journal for October last, there were several interesting cases reported of accidental swallowing foreign substances, some of which were, perhaps, not less remarkable than the very interesting case now reported by Dr. Andrews. In one case a copper cent passed per anum after four months' duration; in other cases, copper cents had been swallowed, and years afterward had not been heard from—they were still retained or passed without knowledge.

Dr. Gibbs continued his remarks nearly as follows: I did not, however, rise to point to a record of similar cases, but to make brief mention of a case of some interest, at least to me, where a foreign substance was accidentally swallowed, and lodged in the œsophagus. About three years since I was called to see a boy, about 6 years of age, who it was supposed, had swallowed a spectacle glass, oval in form and of very large size. I found the boy suffering but little, but on trial, it was observed that he could not swallow without difficulty. Hoping to dislodge it from the œsophagus, I gave an emetic, which operated without bringing away the glass. I gave him a little bread to eat, which he chewed very fine and swallowed without difficulty. Thinking that it was possible the glass had reached the stomach, and having nothing with me with which to explore the œsophagus, I left the patient, requesting that I might be informed should subsequent symptoms indicate that the glass was still in the œsophagus.

Three days after, I was sent for again, and was informed that it was probable that the glass had not yet reached the stomach. An exploration with a probang, showed the foreign substance about two-thirds of the way down the œsophagus. Believing it to be bad practice to force it down, until suitable efforts had been made to withdraw it, I explained my views to the friends and again left the patient, with the promise of returning as soon as I could procure a suitable instrument with which to make the contemplated effort at extraction. The instrument was procured on the second succeeding day. In the interval, a rival practitioner, distinguished more for his mature years and large experience, than for his nice appreciation of courtesy or professional etiquette, called and offered his services, remarking that a man must be a very *inefficient* practitioner who could not force a foreign body from the œsophagus into the stomach, a feat which he had several times performed. With unusual good sense, and with commendable fidelity to myself, the proffered services were declined.

The instrument procured, consisted of a long flexible whalebone rod, with a silver head, having some resemblance to the head and gills of a fish. This head had a jointed attachment to the rod. Aided by my friend Dr. Beardsley, the instrument was introduce into the œsophagus, and pushed carefully down until the head had passed the foreign substance; slightly withdrawing it now, and gently manipulating, it soon became apparent that the edge of the glass was caught in the gill of the instrument, which was now carefully withdrawn, bringing with it, to the great joy of the family, the glass, after five days lodged in the œsophagus. The exact dimensions of the glass I have not at hand, suffice it to say, however, that its circumference was somewhat greater than a cent of the old coinage.

In regard to this case I would simply observe, that death has occasionally resulted from insoluble substances swallowed, of less dimensions than the one in this case lodged in the œsophagus. Hence the propriety of trying to withdraw it, and also, in the absence of distressing symptoms, the propriety delaying decided action, in order to the better accomplishment of the desired end. The result of this case justifies the inference that we should never sacrifice our convictions of *judiciousness*, in the hope of escaping the charge of *inefficiency*.

*Dr. H. W. Barrett* remarked as follows: I do not rise for the purpose of making any remarks in regard to the two interesting cases just reported, but to make a brief mention of a case in which a foreign substance was swallowed, of unusual character, accompanied with alarming symptoms.

Several months since, I was called to see a child, about three years of age, which it was supposed, had swallowed several percussion caps. I found the patient vomiting and purging profusely, and in a state of most alarming prostration. Three of the caps came away by vomiting. In view of the peculiar nature of the case, and its alarming character, I thought it advisable to associate Dr. Axtell with myself in the case. Milk, white of eggs, and stimulants, were the remedies principally brought to bear upon the case. Notwithstanding the evacuations from the bowels were profuse, it was thought advisable to give castor oil, with the addition of a little laudanum. Eleven caps were passed per anum, making fourteen in all that had been swallowed. The patient recovered rapidly.

This case I consider interesting, because of the peculiar character of the substance swallowed. I have no remembrance of having seen a similar case on record. It is also interesting, because of the sudden onset and alarming nature of the symptoms and the rapidity of the recovery.

*D. G. W. Hazeltine* remarked as follows: The interesting cases just mentioned, bring to my mind a case that occurred in the practice of my father. I have no records of the case, and cannot give the particulars in regard to it; suffice it to say, that a child passed into its œsophagus a large and heavy piece of lead, which had been used as a nipple shield. It was removed with a double wire, bent so as to form a hook. The peculiarity of this case is the unusual size and weight of the body lodged in the œsophagus.

*Dr. Hazeltine* moved that Dr. O. C. Gibbs be invited to report the cases which have just been verbally given to the society, for the Buffalo Medical Journal.

It is but just to observe that the above cases have been reported from memory, and it is not impossible that some important particulars have been omitted. I have, however, endeavored to bring out the facts in the cases, in nearly the same language in which they were presented to the society.

